# Assessment of antibody-dependent respiratory burst activity from mouse neutrophils on Plasmodium yoelii malaria challenge outcome

**DOI:** 10.1189/jlb.0513274

**Published:** 2014-02

**Authors:** David Llewellyn, Simone C. de Cassan, Andrew R. Williams, Alexander D. Douglas, Emily K. Forbes, Jaime R. Adame-Gallegos, Jianguo Shi, Richard J. Pleass, Simon J. Draper

**Affiliations:** * The Jenner Institute, University of Oxford, United Kingdom; and; † Liverpool School of Tropical Medicine, Liverpool, United Kingdom

**Keywords:** Fc receptor, vaccine, ROS, merozoite, assay, malaria, MSP1, neutrophil

## Abstract

New tools are required to expedite the development of an effective vaccine against the blood-stage infection with the human malaria parasite Plasmodium falciparum. This work describes the assessment of the ADRB assay in a mouse model, characterizing the functional interaction between antimalarial serum antibodies and FcRs upon neutrophils. We describe a reproducible, antigen-specific assay, dependent on functional FcR signaling, and show that ADRB activity is induced equally by IgG1 and IgG2a isotypes and is modulated by blocking FcR function. However, following immunization of mice with the blood-stage vaccine candidate antigen MSP1_42_, no measurable ADRB activity was induced against PEMS and neither was vaccine efficacy modulated against Plasmodium yoelii blood-stage challenge in γ^−/−^ mice compared with WT mice. In contrast, following a primary, nonlethal P. yoelii parasite challenge, serum from vaccinated mice and nonimmunized controls showed anti-PEMS ADRB activity. Upon secondary challenge, nonimmunized γ^−/−^ mice showed a reduced ability to control blood-stage parasitemia compared with immunized γ^−/−^ mice; however, WT mice, depleted of their neutrophils, did not lose their ability to control infection. Thus, whereas neutrophil-induced ADRB against PEMS does not appear to play a role in protection against P. yoelii rodent malaria, induction of ADRB activity after challenge suggests that antigen targets of anti-PEMS ADRB activity remain to be established, as well as further supporting the observation that ADRB activity to P. falciparum arises following repeated natural exposure.

## Abstract (precis)

Malaria-specific antibody-dependent respiratory burst activity from neutrophils is acquired in mice following infection; however, vaccination with a leading vaccine antigen fails to induce this activity.

## Introduction

Throughout the 20th century, vaccination has proved to be the most successful and cost-effective strategy for fighting disease; however, highly efficacious vaccines against major global health threats, such as malaria, remain elusive [1]. Subunit vaccines against the blood stage of the malaria lifecycle, whereby the parasite undergoes multiple rounds of invasion into the host's erythrocytes, followed by asexual replication, have been a significant focus of preclinical vaccine-development efforts. To date, the vast majority of work has focused on two candidate antigens from the invasive Mz form of the parasite: MSP1 and AMA1; however, the progression of these candidates into clinical trials has provided largely disappointing results [2–5]. The limited number of Plasmodium antigen targets studied to date can be attributed, in some part, to the paucity of available preclinical assays with which candidate antigens can be assessed for use in vaccine candidates [6], as well as limited access to nonhuman primate models of P. falciparum blood-stage infection [7]. The lack of such assays comes from a relatively incomplete understanding of how antibody-mediated protection is conferred in vivo in humans, as well as technical limitations. Whereas it is largely accepted that antibodies are the key effectors of blood-stage immunity [8, 9], the mechanism(s) by which such antibodies act remain widely debated.

Currently the “gold standard” in vitro assay for assessing the effectiveness of vaccine-induced or naturally acquired antibodies against blood-stage parasites (the assay of GIA) measures antibodies' cell-independent ability to neutralize parasites and thus, block their ability to invade or grow within erythrocytes [10–12]. Whereas it is highly likely that antibody GIA-type neutralization is an important effector mechanism for some antimalarial antibodies, vaccine candidates selected on the basis of promising GIA induction have, so far, shown limited efficacy in clinical trials. For example, the highest levels of GIA yet induced in humans by vaccination was reported for an AMA1 protein-based vaccine candidate. In this case, immunized volunteers showed high levels of serum GIA (77% mean at 4 mg/mL purified IgG) but failed to exhibit any significant clinical efficacy against controlled human malaria infection with homologous 3D7 clone parasites [4]. Intriguingly, the same vaccine was reported to induce strain-specific efficacy in a Phase IIb field trial in Malian children [13]; however, the number of 3D7-type parasite infections was small, and it remains unreported as to whether protection was associated with in vitro GIA. Another vaccine based on MSP1 and administered in the same AS02 proprietary adjuvant from GSK failed to show efficacy in a Phase IIb field trial in Kenya [5]. This field of vaccine development has thus been directed largely on the results of GIA assays, with disappointing clinical results. Consequently, there is an increasing realization of the need to develop vaccines that also induce different antimalarial antibody effector functions and an urgent need for the development of new assays to detect such responses.

The ability of cytophilic antibodies to initiate cellular immune responses as a result of Fc-dependent signaling has also attracted attention in the context of antimalarial blood-stage immunity. An assay assessing ADCI describes monocytes as key effectors in antibody-dependent antimalarial cellular activity [14]. FcγRIIa/CD32a and FcγRIII/CD16 signaling activates human monocytes to release TNF-α in response to the opsonization of Mz by cytophilic IgG1 and IgG3 antibodies [15–17]. Polyclonal antibodies that showed ADCI activity in vitro were also reported to confer protection when passively transferred to nonimmune humans [9], although no causal link was formally demonstrated between anti-Mz ADCI and protective outcome. Despite these reports, however, the ADCI assay has been notoriously difficult to reproduce and as a result, has not established itself as a mainstream tool for anti-Mz vaccine candidate antigen screening. Nevertheless, the contribution of FcRs to the mediation of blood-stage malaria immunity should not be discarded. Whereas conflicting reports occur as to the role of FcR-dependent mechanisms in protection against P. yoelii rodent malaria [18, 19], IgG antibody-dependent FcR activity has been shown to play an important role in control of infections by Plasmodium berghei XAT [20] and P. berghei, transgenic for the PfMSP1_19_ [21], whereas the inhibitory FcγRIIb/CD32b is reported to affect Plasmodium chabaudi parasite clearance and disease outcome [22].

Whereas the role of monocytes as effectors of antibody Fc-dependent anti-Mz activity remains under investigation, neutrophils represent an alternative and plausible candidate cell population for clearing blood-stage parasites, given their high phagocytic efficiency and their ability to generate ROS. In particular, a fast clearance of P. falciparum in Gabonese children has been correlated with high ROS production [23]. Additionally, the ADRB assay, which measures neutrophil ROS production in response to opsonized Mz, has been associated recently with protection against clinical malaria in an endemic population [24]. Conversely, assays measuring GIA in the serum of naturally exposed humans have not established a clear association with clinical disease outcome, with studies being divided in their support for a role of GIA in naturally acquired immunity [11]. The association of ADRB with natural clinical protection provides a new opportunity to assess a largely neglected mechanism by which antibodies could be controlling blood-stage malaria infection. Here, we investigated the mechanisms of action underlying ADRB activity in the mouse model and the contribution of ADRB activity in mediating P. yoelii rodent malaria challenge outcome in MSP1-vaccinated and naive, nonimmunized mice.

## MATERIALS AND METHODS

### Animals and immunizations

All procedures were performed in accordance with UK Animals (Scientific Procedures) Act Project License and were approved by the University of Oxford Animal Care and Ethical Review Committee. Six- to 8-week-old WT female BALB/c (H-2^d^), C57BL/6 (H-2^b^), and TO outbred mice were sourced from Harlan UK (Oxon, UK). BALB/c γ^−/−^ [25] and C57BL/6 CD32b^−/−^ (002848; The Jackson Laboratory, Bar Harbor, ME, USA) mice were provided from the Queen's Medical Centre (Nottingham, UK) and bred at the Wellcome Trust Centre for Human Genetics, University of Oxford (UK). Knockout mouse genotypes were confirmed by PCR [26, 27]. Mice were anesthetized with IsoFlo (Abbott Animal Health, Berkshire, UK) before i.m. immunization in a total volume of 50 μL, split equally between each musculus tibialis, unless stated otherwise.

### Vaccines and generation of antigen-specific sera

Human rAdHu5 and MVA vectors expressing the PyMSP1_42_ [28] or the PfMSP1 clinical vaccine construct PfM128 [29] have been described previously. The adenovirus vaccines ifu were measured as described previously [26]. When used to immunize, AdHu5-PyMSP1_42_ at 1.5 × 10^9^ ifu or AdHu5-PfMSP1 at 7 × 10^8^ ifu and MVA at 1 × 10^7^ pfu were formulated in endotoxin-free, low-phosphate PBS (Gibco-Invitrogen, Life Technologies, Paisley, UK) and administered 8 weeks apart (Ad-M). Serum was harvested 14 days following the MVA boost. The use of chimpanzee adenovirus serotype 63 and MVA expressing a transmission-blocking malaria vaccine candidate Pfs25 to raise Pfs25-specific antibody responses in the same manner has been reported previously [30].

PyMSP1_19_-, PyMSP1_33_-, and PfMSP1_19_ (3D7/ETSR allele)-GST fusion proteins, as well as GST control, were produced in an Escherichia coli expression system, as described previously [28, 29]. rPyMSP1_19_, fused to IMX108 (mouse complement C4 binding protein) [31], was kindly provided by Dr. F. Hill (Imaxio, Lyon, France). Generation of immune sera against PyMSP1_19_, Ova, and GST has been described previously. Briefly, BALB/c mice were immunized with recombinant proteins using the following regimes, respectively: three immunizations of 20 μg PyMSP1_19_-GST formulated in AdjuPhos adjuvant (Brenntag Biosector, Frederikssund, Denmark) given at 3-week intervals (PPP) or a single 20-μg PyMSP1_19_-GST in AdjuPhos immunization given 8 weeks after a 1.0 × 10^9^ ifu AdHu5 prime (AP); three immunizations of 20 μg Grade VII Ova (Sigma-Aldrich, Dorset, UK) in available adjuvants at 3-week intervals [32]; or one immunization of 5 μg GST protein in Montanide ISA 720 [33]. Serum was harvested 14–17 days after final immunization.

### P. falciparum Mz lysate

P. falciparum 3D7 clone parasites were maintained routinely in culture, as described previously [34]. The supernatant of a 20-mL in vitro culture of P. falciparum at 10% hematocrit and 10–15% parasitemia was harvested and replaced daily and centrifuged at 830 *g* to pellet and discard RBCs. After 10 days, pooled supernatant was centrifuged at 1500 *g* for 25 min to pellet-free Mz. The Mz pellet was washed twice in PBS and resuspended in 500 μL PBS. The resulting suspension was vortexed vigorously and freeze-thawed in aliquots to form a lysate. Anti-Mz immune sera were generated by immunizing BALB/c mice s.c., once with 50 μL Mz lysate formulated in 50 μL CFA, followed by two further s.c. immunizations of 50 μL Mz lysate in 50 μL IFA at 3-week intervals. Serum was harvested 21 days later.

### Isotype-specific PfMSP1_19_ mAb

PfMSP1_19_-specific chimeric mouse IgG1 and IgG2a mAb against the C1 epitope were expressed in HEK293 and CHO cell lines as described previously [35]. Briefly, cells were cultured in hyperflasks for 17 days in selective media [DMEM supplemented with 10% low IgG FBS (Life Technologies, Carsbad, CA, USA), 1% penicillin/streptomycin (Sigma-Aldrich), 1% L-glutamine (Sigma-Aldrich), 250 μg/mL mycophenolic acid (Sigma-Aldrich), 12.5 μg/mL xanthine (Sigma-Aldrich), 1/1000 vol/vol hygromycin (Sigma-Aldrich; for IgG1), or 1/1000 vol/vol geneticin (Invitrogen, Life Technologies, Paisley, UK; for IgG2a)] before supernatant was collected. mAb IgG was purified from media using protein G drip columns (Sigma-Aldrich), according to the manufacturer's instructions, and IgG concentrations were determined using a ND-1000 spectrophotometer (NanoDrop Technologies, Wilmington, DE, USA).

### Mouse ADRB assay

Two methodologies were used to assess mouse PMN ADRB activity.

#### Recombinant protein-coated plates.

PyMSP1_19_-GST (100 μL) or PfMSP1_19_-GST protein at 10 μg/mL (unless otherwise stated) was adsorbed onto Nunc opaque MaxiSorp 96-well plates at RT overnight. Plates were then washed three times with PBS and blocked for 1 h with casein block solution (Pierce, Fisher Scientific, Loughborough, UK) before a second wash. Serum diluted 1:100 in PBS (100 μL) (unless otherwise stated), or epitope-matched anti-PfMSP1_19_ mouse IgG1 and IgG2a mAb [35] at 8.3 μg/mL were then added and incubated for 1 h at 37°C. Mouse neutrophils were isolated from bone marrow extracted from the femurs and tibias of 6- to 20-week-old BALB/c, C57BL/6, γ^−/−^, and CD32b^−/−^ mice using Percoll (Sigma-Aldrich) density gradients and resuspended in neutrophil buffer (HBSS, 1% glucose, 0.1% BSA) at 1 × 10^7^ PMNs/mL [36]. Purified cells were confirmed to be Ly6C^int^ and CD11b^+^ by flow cytometry, and purity was assessed by Giemsa-stained slides. Within 2 min of a final wash of the assay plate in PBS, 50 μL isoluminol (Sigma-Aldrich; 0.04 mg/mL) and 50 μL mouse cells at 1 × 10^7^ PMNs/mL (unless otherwise stated) were added to each well, and luminescence—in RLU—resulting from ROS, released by PMNs reacting directly with isoluminol, was read each minute for 1 h using a Varioskan Flash luminometer. To investigate the role of FcR signaling in ADRB activity (see Fig. 2B), PMNs were incubated for 15 min at 4°C with rat anti-mouse CD16/32 mAb (clone 93; eBioscience, Hatfield, UK) or control rat IgG (Sigma-Aldrich).

#### PyPEMS in solution.

Two TO mice were inoculated i.p. with 100 μL lethal P. yoelii strain YM pRBCs and monitored until blood-stage parasitemia reached 30–50%. Blood was taken by cardiac puncture and cultured for 24 h at 8% hematocrit and 37°C to allow parasites to mature to late schizonts [37]. Infected cells were then isolated on a 65% Percoll gradient [38], washed, and resuspended in PBS at 1.8 × 10^5^ schizonts/mL. These PEMS were then frozen to lyse the RBCs and release the P. yoelii Mz. ADRB activity was assessed by adding 20 μL PyPEMS and 5 μL neat sera to each well of a one-half area opaque 96-well plate (Pierce, Fisher Scientific) and incubating at 37°C for 1 h before addition of neutrophils and isoluminol as above. For antigen depletions (see Fig. 7), 5 μL serum was incubated with 5 μL PyMSP1_19_-GST or PyMSP1_33_-GST at 1 mg/mL for 1 h at RT before incubation with Mz. This protocol was shown to be effective by ablation of ADRB activity on PyMSP1_19_-GST- and PyMSP1_33_-GST-coated plates by the respective antigen depletions (Supplemental Fig. 1).

For both assays, RLU readouts were indexed against a positive reference serum from Ad-M PfMSP1-immunized, Ad-M PyMSP1_42_-immunized, or twice Py17XNL-challenged mice for the PfMSP1, PyMSP1, and PyPEMS assays, respectively. All readouts are thus calculated as a proportion of a standard positive control (see Results).

### ELISA

Total IgG ELISAs were carried out using a standardized ELISA methodology [39]. AUs were determined by comparison with a standard curve of pooled sera from mice immunized with Ad-M PyMSP1_42_, diluted twofold down the plate starting from a 1:1000 dilution. OD_405_ was read using a BioTek ELx800 microplate reader (BioTek, Bedfordshire, UK). Naive mouse serum samples were negative for antigen-specific responses on all plates (data not shown). Plates were developed until positive control samples reached an OD_405_ of 1.0, and this point was defined as 1 AU, with AU read off of the resulting curve [39]. For mice receiving PPP or AP immunization, serum total IgG end-point ELISAs were carried out as described previously [28]. End-point titers were defined as the dilution at which sample absorbance reached 3 sd greater than the OD_405_ for serum from a naive mouse. A standard, positive serum sample and naive serum sample were included as controls for each assay.

Antigen-specific IgG1 and IgG2a responses were also determined with a standardized ELISA method, as described previously [32]. Briefly, 96-well plates were coated with PyMSP1_19_-IMX108 protein to avoid measuring responses to GST in mice immunized with PyMSP1_19_-GST. Standard curves were made with purified mouse IgG1 and IgG2a mAb (eBioscience) starting at a concentration of 20 μg/mL and diluted threefold. After blocking, test serum was added in duplicate wells and incubated for 2 h before washing. Biotin anti-mouse IgG1 or IgG2a (Becton Dickinson, San Diego, CA, USA) was then added for 1 h, followed by washing and incubation with extravidin alkaline phosphatase (Sigma-Aldrich) for 30 min. Plates were then developed using the same reagents as for total IgG ELISA and isotype units, calculated as for total IgG AU.

### Flow cytometry

Whole mouse blood was collected from tail veins into 200 μL, 10 mM EDTA in PBS and spleens harvested and processed as described previously [40]. Briefly, spleens were crushed and passed through a 70-μm cell strainer before treatment with ACK lysis buffer to lyse RBCs. Whole blood samples were also lysed in a similar manner. After washing, lymphocyte and splenocyte samples were resuspended in 200 μL and 7 mL PBS/BSA, respectively. The resulting cell suspensions (150 μL) were surface-stained for 30 min at 4°C with Alexa Fluor 700-labeled anti-CD11b (clone M1/70), allophycocyanin-labeled anti-Ly6C (clone HK1.4), and PerCPCy5.5-labeled anti-CD8α (clone 53-6.7). Cells were then washed twice in 150 μL PBS/BSA and resuspended in 200 μL PBS/BSA. Samples were run on an LSR II flow cytometer (BD Biosciences, San Jose, CA, USA) with stopping gates set at 100,000 CD8^+^ events for splenocyte samples and 10,000 CD8^+^ events for lymphocyte samples. Granulocytes were gated by forward- and side-scatter and neutrophils identified as the CD11b^+^ Ly6C^int^ granulocyte population (Supplemental Fig. 2). Data were analyzed using FlowJo v8.8.7.

### PMN^−^

To deplete neutrophils, mice were injected i.p. with 0.5 mg 1A8 rat mAb (Bio X Cell, West Lebanon, NH, USA) [41, 42], 1 day before and 3 days after challenge with P. yoelii pRBCs. Control animals were given 0.5 mg control rat IgG (Sigma-Aldrich) i.p. at the same time-points. Depletion was monitored at 1 and 7 days after challenge in the blood and spleen of an additional group of animals, as above by flow cytometry. The percentage depletion attained was calculated using the number of PMNs (Ly6C^int^ CD11b^+^ granulocytes; Supplemental Fig. 2B) per lymphocyte (Supplemental Fig. 2A) in a mouse treated with control rat IgG compared with the number of PMNs/lymphocyte in a 1A8-treated mouse as follows:

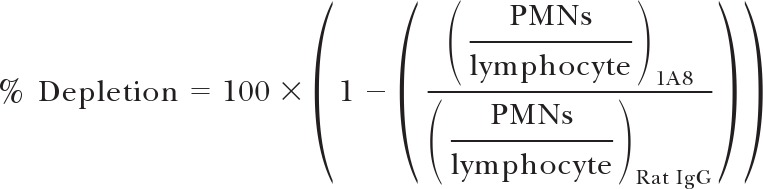


### P. yoelii challenge

Lethal (strain YM) and nonlethal (strain 17XNL) P. yoelii challenges were carried out as described previously [28]. Experimental animals were infected by i.v. injection with 10^4^ or 10^6^ pRBCs. Blood-stage parasitemia, calculated as percentage of infected RBCs, was monitored from Day 3 postchallenge by microscopic examination of Giemsa-stained thin blood smears. Mice whose blood smears had no observable parasites in 50 fields of view were considered uninfected, and those that reached the humane end-point of 50%-infected RBCs were culled.

### Statistical analysis

All statistical analysis was carried out using Prism v.5.03 (GraphPad Software, La Jolla, CA, USA). Comparisons between two groups were conducted using Mann-Whitney test or Wilcoxon matched-pairs signed-rank test when data were paired. Comparisons among three or more groups were assessed by means of a Kruskal-Wallis test (independent groups) or a Friedman test (paired data/repeated measures of samples). Post hoc Dunn's multiple comparison tests were used to identify contributing factors to significant Kruskal-Wallis or Friedman tests. Two-way ANOVAs were used to assess intra- and interassay variability by analyzing the effect of sample and that of assay run respectively, as well as determining each factor's relative contribution to measured *R*^2^. Parasitemia was analyzed using an AUC analysis, and correlations between ELISA titer and ADRB activity were tested using Spearman rank correlation. Statistical significance was considered at *P* ≤ 0.05.

## RESULTS

### ADRB assay development

**Figure 1.**
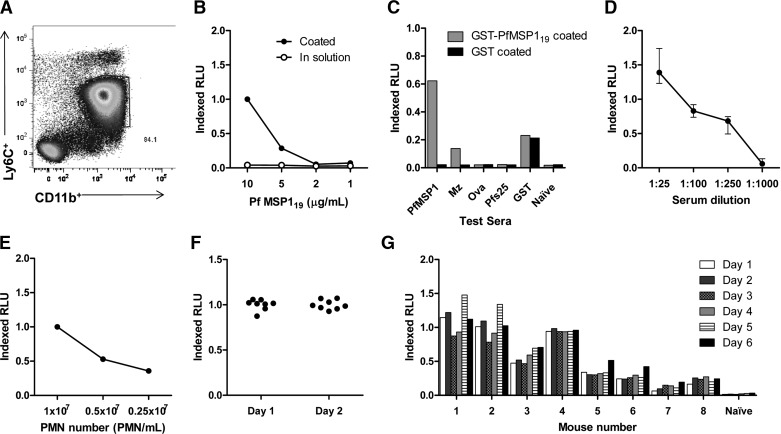
Protein ADRB assay optimization. Assay variables were assessed using serum from Ad-M PfMSP1-immunized BALB/c mice and neutrophils enriched from the bone marrow of naive BALB/c mice. Plates were coated with 10 μg/mL PfMSP1_19_-GST protein, incubated with serum diluted 1:25 in PBS, and assayed with 50 μL neutrophils/well at 1 × 10^7^ PMNs/mL unless stated otherwise. (A) Representative flow plot of granulocyte cell preparation used in the assay after enrichment for neutrophils (also see Supplemental Fig. 2). (B) ADRB activity induced by immune sera against PfMSP1_19_-GST protein in solution or coated onto a plate at decreasing concentrations. (C) The assay plate was coated with GST or PfMSP1_19_-GST recombinant protein at 10 μg/mL. Bars indicate ADRB (all indexed against PfMSP1-positive sera on a PfMSP1_19_-GST-coated plate) induced by sera, taken from mice immunized with PfMSP1, P. falciparum Mz in Freund's adjuvant (Mz), Ova, Pfs25, GST, or with no prior immunization (Naïve). (D) ADRB induction by anti-PfMSP1 sera at increasing serum dilutions (points represent mean ± sem of six independent samples) and (E) at a constant serum dilution of 1:100 with decreasing numbers of cells/well (50 μL cells added at presented cell number/mL). (F) Serum pooled from six Ad-M PfMSP1-immunized mice was tested in eight replicate wells on 2 days to assess intra-assay variability. (G) Serum from eight Ad-M PfMSP1-immunized mice and one naive mouse was tested on 6 separate days in the assay to assess interassay variability. In all panels, bars, and points represent mean of two assay replicates for each sample unless stated otherwise.

Neutrophil respiratory burst activity (NADPH oxidase activation), induced by mouse sera, was assessed initially using neutrophils enriched from mouse bone marrow and recombinant protein coated onto a plate, according to published methodologies [19, 43]. To establish the assay, sera from BALB/c mice, immunized with Ad-M PfMSP1, were used, which were known to be reactive against PfMSP1_19_-GST protein [29]. Percoll separation was used to enrich neutrophils to >95%, as determined by examination of Giemsa-stained slides. The isolated cell population was confirmed as Ly6C^int^ CD11b^+^ granulocytes by flow cytometry (**Fig. 1A**). Results in a previous study were indexed according to a positive reference serum, which was included each time the assay was run [24]. This approach was also applied here, using a pool of positive antigen-specific serum (see Materials and Methods). As such, results are presented in iRLU, where

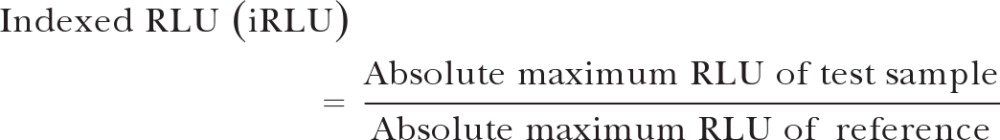


Respiratory burst activity was induced when the rPfMSP1_19_ antigen was coated onto the assay plate at a concentration ≥5 μg/mL, whereas the same concentrations of antigen present in solution failed to induce a response (Fig. 1B). Coated protein likely forms an array, not present when the antigen is in solution, which causes FcR on the neutrophil surface to colocalize and thus, initiate activatory γ-chain signaling and the respiratory burst [44–46]. After coating separate wells of the assay plate with two different antigens (GST and PfMSP1_19_-GST), respiratory burst activity, induced by a panel of different sera, was tested. The panel of sera included five different samples from mice immunized with Ad-M PfMSP1 [29], P. falciparum Mz in Freund's adjuvant (thus containing PfMSP1_19_, as well as many other antigens), Ova [32], a transmission-blocking malaria vaccine candidate Pfs25 [30], and GST, as well as serum from nonimmunized mice (naive). Positive ADRB activity was induced by sera only when the immunization antigen matched the antigen coated onto the assay plate (Fig. 1C), confirming the specificity of the assay. Additionally, the magnitude of the iRLU response decreased with decreasing coating antigen concentration (Fig. 1B), increasing serum dilutions (Fig. 1D), and reduced cell numbers in each well (Fig. 1E). As such, a set of assay conditions was established for further experiments, as follows: antigen coated on the assay plate at 10 μg/mL, serum diluted 1:100 in PBS, and 50 μL neutrophils added to each well at 1 × 10^7^ PMNs/mL. The use of maximal assay conditions with a mid-range serum dilution (1:100) ensured that both higher and lower assay responses could be detected for test sera. With the use of these assay conditions and a PfMSP1_19_-GST-coated plate, assay variability was also determined. To assess intra-assay variability, a single pool of serum derived from eight Ad-M PfMSP1-immunized BALB/c mice was tested for induction of ADRB activity in eight separate wells with neutrophils from a single donor mouse on each of 2 days. Interassay variability was determined by assessing ADRB activity elicited by serum from each of the eight Ad-M PfMSP1-immunized BALB/c mice on 6 different days (i.e., with a different neutrophil donor each day). Whereas intra-assay variability was not significant (two-way ANOVA *F*_7,7_=0.70, *P*=0.67; Fig. 1F), there was significant interassay variability (two-way ANOVA *F*_5,54_=23.08, *P*<0.0001; Fig. 1G), although assay repeat (or different neutrophil donor) accounted for <5% of the observed variance compared with >93%, resulting from differences among the vaccine response in the test samples, as determined by *R*^2^ analysis.

### Role of FcR-mediated pathways in ADRB induction

**Figure 2.**
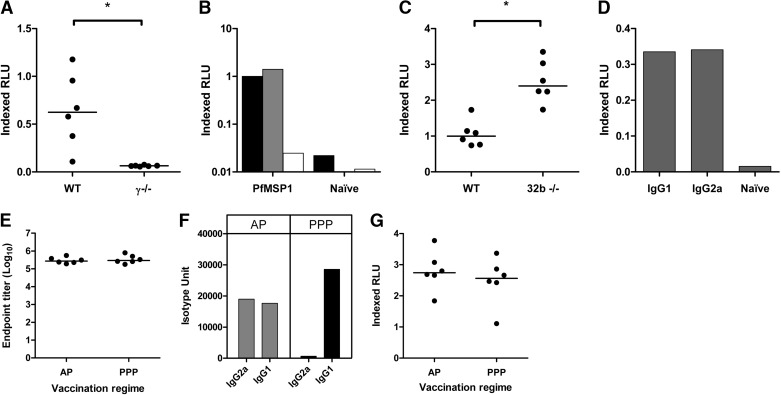
Role of FcR-mediated pathways in ADRB induction. Serum was collected from BALB/c and C57BL/6 mice immunized with Ad-M PyMSP1_42_ or BALB/c mice immunized with three doses of PyMSP1_19_-GST with AdjuPhos (PPP) or one dose AdHu5-PyMSP1_42_, followed by one dose PyMSP1_19_ with AdjuPhos (AP). Assays were set up with 10 μg/mL PyMSP1_19_-GST-coated plates, 50 μL/well neutrophils at 1 × 10^7^ PMNs/mL and serum diluted 1:100 in PBS run in duplicate wells. Neutrophils were isolated from the bone marrow of WT BALB/c and C57BL/6 mice, plus γ^−/−^ and CD32b^−/−^ mice on the same genetic backgrounds, respectively. ADRB induction was assessed by: (A) BALB/c and γ^−/−^ PMNs in response to sera from BALB/c mice immunized with Ad-M PyMSP1_42_; (B) BALB/c PMNs in response to sera from Ad-M PfMSP1-immunized or naive BALB/c mice (on a PfMSP1_19_-GST-coated plate), where PMNs had been preincubated with neutrophil buffer (black), control rat IgG (gray), or anti-CD16/32 mAb (white); (C) C57BL/6 and CD32b^−/−^ PMNs in response to sera from C57BL/6 mice immunized with Ad-M PyMSP1_42_; and (D) BALB/c PMNs in response to mouse isotype-specific chimeric anti-PfMSP1_19_ mAb at 8.3 μg/mL (on a PfMSP1_19_-GST-coated plate). For mice immunized with AP and PPP regimes, (E) PyMSP1_19_ end-point ELISA titers against PyMSP1_19_-IMX108-coated plates, (F) IgG1 and IgG2a isotype-specific ELISA titers against the same protein as total IgG, and (G) ADRB activity using BALB/c PMNs were determined. *P < 0.05 (Wilcoxon matched-pairs signed-rank test). Bars and points represent means of two replicates for each sample, and medians on dot plots are shown, represented by lines.

The role of FcR-mediated pathways in inducing ADRB activity was investigated using γ^−/−^ and FcγRIIb knockout (CD32b^−/−^) mice. The number of neutrophils added to each well was kept constant between groups by counting the number of cells using a hemocytometer and assessing the percentage purity of neutrophils by Giemsa-stained slide, before adjusting accordingly. In this case, ADRB activity was measured against PyMSP1_19_-GST protein (using serum from mice immunized with PyMSP1-based vaccines). The response induced by neutrophils from WT mice in response to serum of BALB/c mice, immunized i.m. with Ad-M PyMSP1_42_ (median=0.62 iRLU), was ablated when using neutrophils enriched from the bone marrow of γ^−/−^ mice (median=0.06, *P*=0.03; **Fig. 2A**). Additionally, preincubation of WT neutrophils with anti-CD16/32 mAb ablated their ability to induce ADRB activity (Fig. 2B). On the other hand, neutrophils isolated from the bone marrow of C57BL/6 mice lacking the inhibitory FcγRIIb were able to induce higher ADRB (median=2.40 iRLU) than those isolated from WT mice (median=1.00 iRLU, *P*=0.03; Fig. 2C). Thus, it appears that ADRB activity is dependent on FcR-mediated pathways; in particular, common γ-chain signaling is necessary while regulation occurs via CD32b. Furthermore, given that mice lack CD32a [47–49] (the activatory FcγRII) and given the ablation of ADRB with anti-CD16/32 mAb, CD16/FcγRIII appears likely to mediate ADRB induction in the mouse system.

### Role of IgG isotypes in ADRB induction

To determine the contribution of different IgG isotypes to ADRB induction, chimeric mouse IgG1 and IgG2a mAb against the C1 epitope of PfMSP1_19_ were expressed in HEK293 and CHO cell lines, respectively [35]. Purified antibodies of each isotype at 8.3 μg/mL induced indistinguishable levels of ADRB activity against PfMSP1_19_-GST (Fig. 2D). Additionally, mice immunized i.m. with a PyMSP1_42_ adenovirus prime PyMSP1_19_-GST protein-in-AdjuPhos boost regime (AP) or a three-dose PyMSP1_19_-GST protein-in-AdjuPhos regime (PPP) [32] induced the same total IgG titer against PyMSP1_19_, as measured by end-point ELISA (Fig. 2E) but different profiles of IgG isotypes (Fig. 2F). AP immunization resulted in a balanced isotype response with equivalent induction of IgG1 and IgG2a. In contrast, PPP immunization resulted in an IgG1-dominated response with negligible levels of IgG2a being induced, as shown previously for the AdjuPhos adjuvant [32]. ADRB activity was assayed using serum from mice immunized with these two vaccination regimes, and no difference was observed (*P*=0.39; Fig. 2G), in agreement with the epitope-matched chimeric mAb. These data suggest that the mouse IgG1 and IgG2a isotypes can both elicit equivalent ADRB activity from neutrophils.

### FcRs and efficacy against P. yoelii challenge

**Figure 3.**
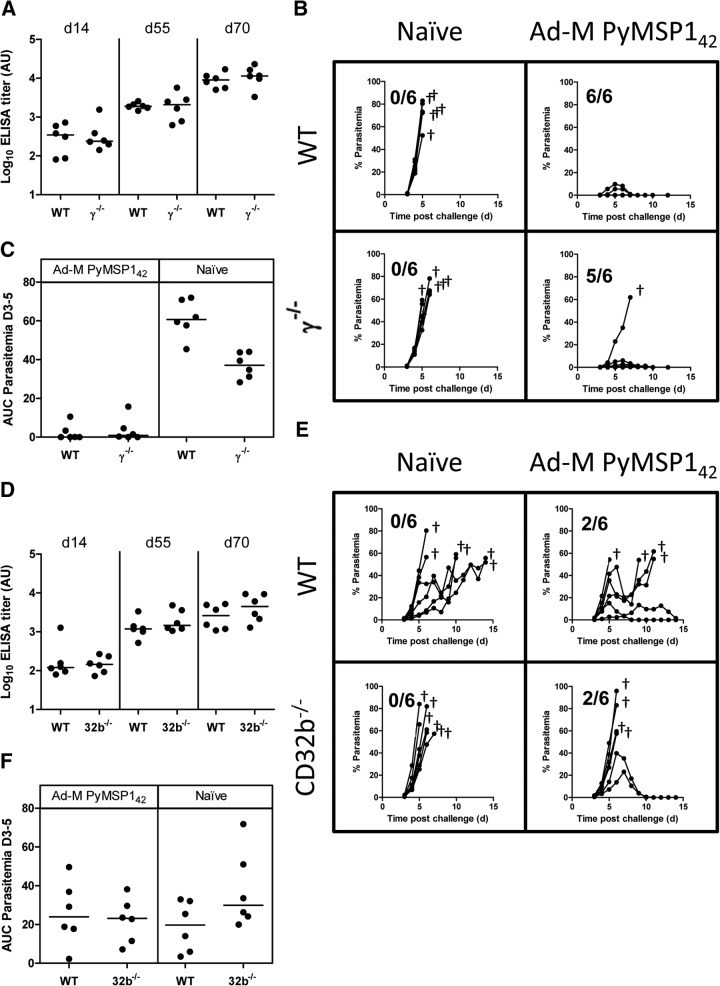
P. yoelii challenge of γ^−/−^and CD32b^−/−^ mice. Challenge outcome in BALB/c, γ^−/−^, C57BL/6, and CD32b^−/−^ mice was investigated. All mice (*n*=6/group) were immunized i.m. with Ad-M PyMSP1_42_. PyMSP1_19_ ELISA titers were determined in (A) BALB/c and γ^−/−^ mice and (D) C57BL/6 and CD32b^−/−^ at 14, 55, and 70 days post adenovirus immunization (MVA boost occurred on Day 56). At Day 70, all mice were challenged i.v. with 10^4^ P. yoelii YM pRBCs. Blood-stage parasitemia is reported as the percentage of infected RBCs over time in (B) BALB/c and γ^−/−^ groups and (E) C57BL/6 and CD32b^−/−^ groups. AUC analysis for Days 3–5 (D3–5) was conducted on (C) parasitemia plots for BALB/c and γ^−/−^ mice and (F) plots from C57BL/6 and CD32b^−/−^ mice. Lines on dot plots represent median values. †Animal being culled after reaching the humane end-point of 50% blood-stage parasitemia.

One of the goals of using this assay was to assess the ability of activity measured in the assay to associate with protective efficacy against blood-stage malaria infection. The previous data showed that ADRB activity was dependent on FcR signaling in the mouse model. Given that it is well-established that PyMSP1-based vaccine efficacy in the P. yoelii blood-stage challenge model is antibody-mediated [28, 50], the impact of FcR modifications on challenge outcome in vaccinated and P. yoelii-infected mice was assessed. Vaccine doses were chosen based on prior experience with this model to allow for improved or reduced vaccine efficacy to be observed. Immunization i.m. with Ad-M PyMSP1_42_ induced the same levels of anti-PyMSP1_19_ antibodies in WT and γ^−/−^ BALB/c mice at all time-points (**Fig. 3A**). The presence or absence of FcR signaling thus had no impact on viral vectored vaccine antibody immunogenicity. All mice were challenged subsequently with 10^4^ lethal P. yoelii strain YM pRBCs. The rate of survival was similar between WT and γ^−/−^ groups in vaccinated and naive, nonimmunized control groups (Fig. 3B). AUC analysis of Days 3–5 parasitemias for vaccinated mice showed no difference in parasite burden between WT and γ^−/−^ groups (*P*=0.31), although naive mice showed a higher parasite burden in the WT group (*P*=0.002; Fig. 3C), indicating a small but significant delay in the knockouts reaching end-point parasitemia. Similarly, in WT and CD32b^−/−^ C57BL/6 mice, antibody levels induced by vaccination did not differ at any time-point between groups (Fig. 3D), nor was there any difference in challenge outcome survival rates (Fig. 3E) or parasite burden (AUC analysis for Days 3–5) for vaccinated (*P*=0.93) or naive groups (*P*=0.13; Fig. 3F).

Thus, despite the inability of γ^−/−^ mice to induce ADRB, the vaccinated mice were still able to control parasitemia, indicating that vaccine-induced efficacy in this model is unlikely ADRB- or FcR-dependent. Likewise, the ability of CD32b^−/−^ mice to induce higher ADRB did not enhance vaccine-mediated control of parasitemia. Taken together, all of these initial protection data suggested that FcR-mediated effector functions (including ADRB activity, as measured in vaccinated mice) are not essential in determining the outcome of primary challenge in the murine P. yoelii model, neither in vaccinated nor control mice.

### Assaying ADRB with coated antigen versus whole Mz

The ADRB assay methodology established above is not limited to use with the MSP1_19_ antigen but can be used to assess functional antibody activity against any antigen under study once coated onto the plate [43, 51]. It also does not permit assessment or comparison of the effectiveness of ADRB induction by antibodies binding to different antigens, as presented in the context of the intact Mz itself. We therefore decided to assess the possibility of using whole malaria parasites in the assay rather than an array of rMSP1_19_ protein. Whreas the vaccines used above induce serum antibodies that show ADRB responses against MSP1_19_ protein coated onto the plate, it was wholly possible that the same vaccines do not induce functional ADRB from neutrophils via opsonization of Mz. The first challenge dataset supported this hypothesis, indicating that there was no association between ADRB activity, as measured in the assay against PyMSP1_19_ (Fig. 2A and C) and vaccine efficacy. As such, the assay was established to assess ADRB activity in response to P. yoelii YM Mz (PyPEMS) and was shown to be reproducible in the same fashion as the PyMSP1_19_ assay (Supplemental Fig. 3).

**Figure 4.**
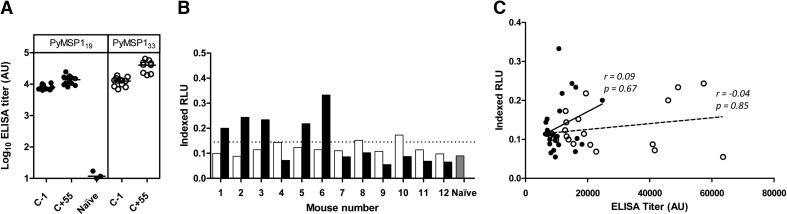
PyMSP1 ELISAs and PyPEMS ADRB. Twelve WT BALB/c mice were immunized i.m. with Ad-M PyMSP1_42_ before being challenged i.v. with 10^6^ P. yoelii 17XNL pRBCs. (A) PyMSP1_19_ (●) and PyMSP1_33_ (○) antibody titers were assessed by standardized ELISA in mice, 2 weeks after boost (1 day before challenge; C−1), and 55 days after challenge (C+55). PyMSP1_19_ titers for serum from three naive, control mice are also shown. (B) ADRB activity induced by BALB/c PMNs (1×10^7^ PMN/mL) against PyPEMS was determined using sera (neat) from the C−1 (white bars) and C+55 (black bars) time-points. Background cutoff, defined as 3 sd above the mean of the level of ADRB activity induced by naive BALB/c mouse serum (*n*=4) in the same assay (gray bar), is indicated by the dotted line. (C) PyMSP1_19_ (●) and PyMSP1_33_ (○) ELISA titers were plotted against pre- and postchallenge anti-PyPEMS ADRB activity. Lines on dot plots represent medians, bar charts represent mean of two assay replicates, and Spearman rank correlations (*r*) shown on scatter plot for PyPEMS ADRB with PyMSP1_19_ (solid line) and PyMSP1_33_ (broken line) ELISA titers.

To investigate whether anti-PyPEMS ADRB was induced by immunization, 12 WT BALB/c mice were first immunized i.m. with Ad-M PyMSP1_42_ and then challenged with a nonlethal strain of P. yoelii (17XNL). Two weeks after the boost (time of challenge), sera from immunized mice showed PyMSP1_19_- and PyMSP1_33_-specific antibody responses, as measured by standardized ELISA. These responses were boosted slightly after challenge for PyMSP1_19_ (*P*=0.002) and PyMSP1_33_ (*P*=0.004; **Fig. 4A**). However, following vaccination, the sera did not lead to a significant induction of ADRB activity against PyPEMS (Fig. 4B, white bars). In contrast, following challenge with 10^6^ Py17XNL pRBCs and parasite clearance, serum from five of 12 mice did induce detectable levels of ADRB activity against P. yoelii Mz (Fig. 4B, black bars).

This induction of ADRB activity could be a result of boosting pre-existing antibody titers against PyMSP1_42_ or an induction of antibody responses against de novo parasite antigens. It seemed more likely that the resultant activity is a result of recognition of new antigens, as there was no correlation between PyMSP1_19_- or PyMSP1_33_-specific total IgG ELISA titer and anti-Mz ADRB activity after challenge (*r*=0.44, *P*=0.15; *r*=0.07, *P*=0.86, respectively) or when the pre- and postchallenge data were pooled (PyMSP1_19_: *r*=0.09, *P*=0.67; PyMSP1_33_: *r*=−0.04, *P*=0.85; Fig. 4C).

### ADRB activity and secondary parasite exposure

**Figure 5.**
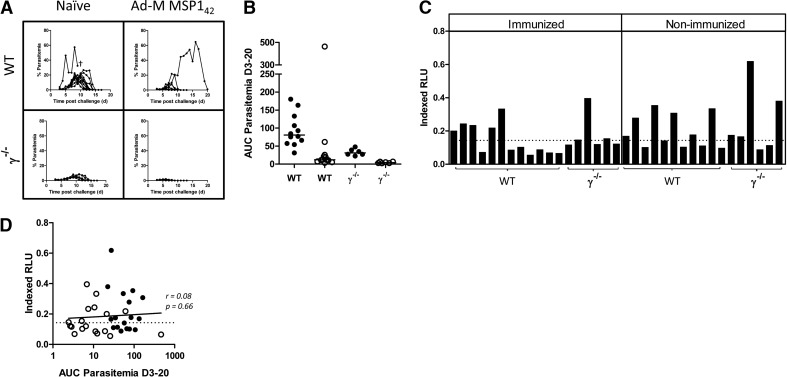
ADRB and P. yoelii malaria challenge. BALB/c or γ^−/−^ mice were immunized i.m. with Ad-M PyMSP1_42_ and challenged 2 weeks later with 10^6^ P. yoelii 17XNL pRBCs. (A) Blood-stage parasitemias in BALB/c and γ^−/−^ groups are reported as the percentage of infected RBCs. (B) AUC analysis of parasitemia between Days 3 and 20 was conducted for naive/nonvaccinated (●) and immunized (○) groups. (C) Fifty-five days after challenge, ADRB activity of mouse serum against PyPEMS was assessed as before. (D) Postchallenge anti-PyPEMS ADRB activity was plotted against AUC, calculated between Days 3 and 20. Lines on dot plots represent medians, bar charts represent mean of two replicates for each sample, and line on scatter plot represents Spearman rank correlation. †Animal being culled after reaching the humane end-point of altered behavior due to treatment.

The above data suggested that functional anti-Mz ADRB activity was detectable in the P. yoelii model following vaccination and a nonlethal primary parasite exposure. We therefore sought to assess whether anti-Mz ADRB activity could contribute to protective efficacy following secondary parasite exposure, given the activity in serum was now detectable. To address this, in addition to the 12 immunized WT mice described in Fig. 4, six γ^−/−^ mice were immunized and challenged with nonlethal parasites in the same manner, whereas a matching 18 mice were challenged without prior immunization (**Fig. 5A**). Following primary challenge, AUC analysis of blood-stage parasitemia demonstrated that vaccination achieved significant protection in the WT (*n*=12 vs. 12; *P*=0.001) and γ^−/−^ (*n*=6 vs. 6; *P*=0.004) groups. Intriguingly, γ^−/−^ mice also controlled parasitemia better than WT mice in vaccinated (*P*=0.004) and naive (*P*=0.002) groups (Fig. 5B). On Day 55, after the primary P. yoelii 17XNL challenge (between 35 and 49 days postparasite clearance) and in agreement with the previous observation, serum from 18/35 mice elicited positive ADRB activity against P. yoelii YM Mz (when tested with neutrophils from WT mice; Fig. 5C). The induction of this serum activity was comparable and equally distributed across the immunized and nonimmunized groups, again suggesting that this activity was independent of PyMSP1_42_ vaccine-induced responses. We also hypothesized that ADRB induction may occur as a result of accumulative parasite exposure, in which case, ADRB activity against P. yoelii Mz would be expected to correlate with parasite exposure, as measured by AUC analysis of blood-stage parasitemia; however, no such correlation was seen (*r*=0.08, *P*=0.66; Fig. 5D).

**Figure 6.**
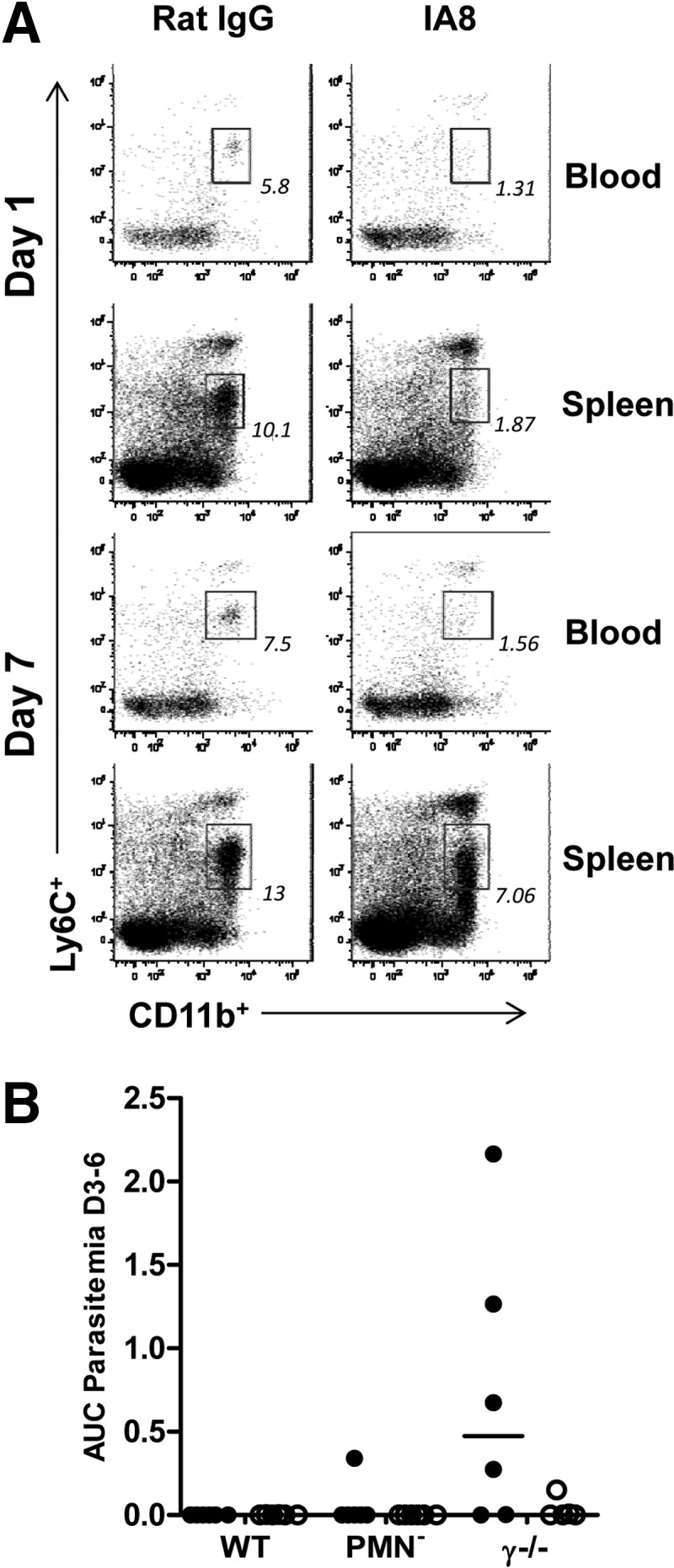
The role of common γ-chain signaling and neutrophils in protection against secondary exposure to P. yoelii. BALB/c or γ^−/−^ mice were immunized i.m. with Ad-M PyMSP1_42_, challenged at Week 10 with 10^6^ P. yoelii 17XNL pRBCs and then rechallenged at Week 18 with 10^6^ P. yoelii YM pRBCs. One day before and 3 days after secondary challenge, six BALB/c and six γ^−/−^ mice were given 0.5 mg 1A8 i.p. (PMN^−^), and all other mice—WT and γ^−/−^—were given 0.5 mg i.p. control rat IgG. (A) Representative flow plots showing granulocytes from the blood and spleen of BALB/c mice treated in parallel with challenged mice on Days 1 and 7 following 1A8 or control rat IgG treatment. Plots thus represent inferred PMN^−^ in challenged mice. Flow data acquisition was normalized by setting stopping gates at 10^4^ or 10^5^ CD8^+^ cells for blood and spleen samples, respectively. Boxes and numbers indicate the neutrophil population and their percentage make-up of the granulocyte population. (B) AUC analysis of parasitemia for Days 3–6 following secondary challenge of nonimmunized (●) and immunized (○) groups. Lines on dot plot represent medians.

To ascertain whether an induction of anti-Mz ADRB activity had any effect on challenge outcome, all 35 mice were rechallenged 8 weeks after the initial nonlethal challenge with 10^6^ lethal P. yoelii YM pRBCs. One day before challenge and 3 days after challenge, one group of six mice in each of the immunized and nonimmunized WT groups was injected i.p. with 0.5 mg 1A8 mAb, a PMN^−^ agent [41, 42]. All other mice received a comparable dose of control rat IgG at the same time-points. PMN^−^ was confirmed in the blood and the spleen of additional control mice at Days 1 and 7 postchallenge by flow cytometry. The blood neutrophil population was depleted by 90% over this time-course. On Day 1 postchallenge, a high level of depletion was visible in the spleen (>80%); however, this population had largely reappeared by Day 7 (**Fig. 6A**).

Following lethal secondary challenge, 66% of all mice were sterilely protected, with no parasites being observed in the blood over the course of the monitoring period. There was no difference in parasite burden among the three immunized groups (WT, PMN^−^, and γ^−/−^), as determined by one-way ANOVA (*P*=0.40; Fig. 6B), although it may remain impossible to ascertain potential differences in the face of such high levels of protective efficacy afforded by the preceding vaccination and P. yoelii 17XNL challenge. On the other hand, there was a significant difference among nonimmunized groups (*P*=0.008), as a result of a greater parasite burden experienced by γ^−/−^ mice than nonimmunized WT mice, as measured by AUC between Days 3 and 6. PMN^−^ mice, in contrast, experienced a similar infectious burden to nondepleted WT mice (Dunn's multiple comparison test; Fig. 6B). It should also be noted that all mice that received 1A8 treatment had completely cleared and/or prevented parasitemia by Day 6 (during the time of maximum PMN^−^). In fact, only two of 11 mice treated with 1A8 developed any detectable parasitemia following secondary challenge.

Thus, overall, it appears that the FcR common γ-chain signaling may play a role in the control of parasitemia during secondary infection of some mice that did not receive a prior effective vaccine, although this control is not mediated by neutrophils. It should also be noted that the γ^−/−^ mice here, experiencing reduced control of secondary parasitemia, exhibited the lowest levels of parasite exposure (and the highest degree of innate protection) in the preceding P. yoelii 17XNL challenge (Fig. 5A and B). However, the induction of serum anti-PyPEMS ADRB activity postchallenge (Fig. 5C) was comparable with the WT-nonimmunized mice, suggesting similar induction of antimalarial responses in these mice, despite the slightly lower parasite burden in the primary challenge.

**Figure 7.**
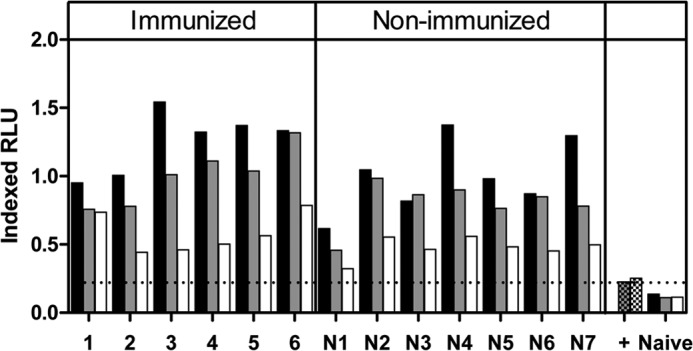
Contribution of anti-PyMSP1_42_ antibodies to post-secondary challenge anti-PyPEMS ADRB activity. BALB/c or γ^−/−^ mice were immunized i.m. with Ad-M PyMSP1_42_ and challenged as in Fig. 6. Fourteen days postsecond challenge, anti-PyPEMS ADRB activity was assessed in mouse serum preincubated with PBS (no depletion; black) or depleted of PyMSP1_19_ (gray)- or PyMSP1_33_ (white)-specific antibodies. Checkered bars (+, positive reference serum) represent background ADRB activity of depleted sera without any antigen coated onto the plate. Results with naive BALB/c mouse serum are also shown.

Despite the lack of association between ADRB activity and challenge outcome in this model, we also assessed ADRB activity following the secondary P. yoelii exposure in the 13 mice showing the highest ADRB response after primary challenge. Serum ADRB activity was enhanced by parasite exposure (**Fig. 7**), showing higher levels in comparison with after primary challenge (Fig. 5C). To address antigen-specificity further in the assay, we depleted sera using the rPyMSP1 proteins (Fig. 7). As expected, assays using plates coated with rPyMSP1_19_ or PyMSP1_33_ protein demonstrated an antigen-specific effect of antibody depletion upon ADRB activity (Supplemental Fig. 1). When tested using PyPEMS, depletion of PyMSP1_19_ antibodies did not significantly decrease the sera's ability to induce ADRB activity (Friedman test; Dunn's multiple comparison test); however, PyMSP1_33_ antibody depletion reduced ADRB activity against PyPEMS similarly in immunized and nonimmunized mice, suggesting that after secondary challenge, the PyMSP1_33_ region of the PyMSP1 antigen is a target of ADRB-inducing antibodies.

## DISCUSSION

The blood-stage malaria vaccine field has often struggled as a result of the lack of in vitro assays with which to screen and assess possible subunit vaccine candidate antigens. Whereas IgG responses to Mz antigens have, in some cases, been correlated with clinical immunity following natural malaria exposure [52–56], little has been done to characterize the functional mechanisms of these antibodies. It is important to note the distinction between effector mechanisms, which may act against the invasive Mz form of the parasite, versus subsequently infected RBCs, which express parasite-derived proteins on their surface. Phagocytic uptake of the latter has been widely described and assayed [57]; however, in the case of P. falciparum vaccine development, such RBC surface antigens are widely thought to be too polymorphic (in most instances) to be realistic vaccine targets. In the context of anti-Mz immunity, the assays of GIA and ADCI have been described previously [11, 17], along with assays of Mz phagocytosis using THP-1 [58] cells or neutrophils [59]. The relationship of the latter with clinical outcome in naturally exposed individuals remains to be established. The assay of ADRB activity using human neutrophils has been reported to associate with clinical immunity in an endemic population in Senegal [24]. Here, we report on an assessment of the ADRB assay in the mouse model and apply it, for the first time, to the field of malaria vaccine development.

Initial work confirmed antigen specificity and established the assay parameters when using recombinant antigen arrayed on the plate. These assay conditions used a minimal volume of serum and allowed for an increase and decrease in ADRB activity to be observed with minimal intra-assay variability. Although some interassay variability was noted when repeatedly using the same sample on freshly isolated PMNs, the inherent differences among individual samples were greater and thus, reliably observed among assay replicates. This mouse ADRB assay also allowed for the roles of FcR-mediated antibody signaling to be assessed. Mice have three classes of activatory FcγRs (I, III, IV) that signal through the common γ-chain ITAMs [47]. The work reported here has shown a dependency on these receptors for ADRB induction, given ablation of this activity when using PMN from γ^−/−^ mice. More specifically, we conclude that ADRB activation is FcγRIII (CD16)-mediated, given that FcγRI (CD64) is not constitutively expressed on mouse neutrophils [60, 61] and thus, is unlikely to contribute to ADRB [a result supported by the ablation of ADRB activity by a CD64-irrelevant mAb (anti-CD16/32)] and that mice lack the activatory FcγRIIa (CD32a) [47, 49]. ADRB induction, however, is not only induced by CD16 but also, is regulated by FcγRIIb (CD32b), given that enhanced levels of activity were observed when using PMN from CD32b^−/−^ mice. It is unlikely that CD32b regulation results from interference with other antibody-FcR binding but rather, results from intracellular ITIM signaling, which initiates the recruitment of Src homology 2 domain-containing inositol phosphatase and impedes CD16 signal transduction [62–64].

The human IgG1 and IgG3 cytophilic isotypes have been reported to be essential for the induction of ADCI activity against the P. falciparum parasite and specific antigens such as MSP3 [15, 65, 66], whereas a human IgG1 mAb against PfMSP1_19_, but not an epitope-matched IgA, has been reported to protect against transgenic P. berghei in humanized mice [21, 67]. Similarly, the cytophilic mouse isotype IgG2a has been reported to be important for protective immunity against blood-stage P. berghei [68] and P. yoelii [69, 70]. However, in our hands, the IgG1 and IgG2a isotypes appeared comparable in their ability to induce ADRB activity from PMN—either using serum skewed toward specific isotype profiles by use of different vaccine immunization regimes [32] or by using chimeric epitope-matched mAbs [35]. We were unable to express sufficient quantities of chimeric epitope-matched mouse IgG2b and IgG3 (data not shown), and so, it remains to be determined whether these isotypes could also function in a similar manner. Notably, mAb of both of these isotypes have been shown to afford efficacy against blood-stage P. yoelii [19, 71]. However, given that mouse IgG1 and IgG2a are reported to signal via CD16 [47, 72] and tend to be the dominant IgG isotypes in serum, these data suggest that total antigen-specific IgG antibody titer in mice is likely to correlate closely with ADRB induction.

In addition to using the ADRB assay to assess antibody-receptor interactions, mouse malaria models provide an invaluable tool for assessing protective outcome. Initial studies indicated that serum from PyMSP1_42_-vaccinated mice could reliably induce ADRB activity from neutrophils in the assay when rPyMSP1_19_ antigen was arrayed on the plate. Despite neutrophils from γ^−/−^ mice lacking the ability to produce ROS in the context of the ADRB assay and CD32b^−/−^ mouse neutrophils having an increased ability to produce ROS, no difference was seen in primary P. yoelii challenge outcome following immunization of these mice (which mounted comparable immune responses with WT controls in response to viral-vector immunization, as seen in a previous study [26]). We also observed little difference in the protective outcome between nonimmunized WT and CD32b^−/−^ C57BL/6 mice following lethal P. yoelii challenge, unlike that reported for P. chabaudi infection on the BALB/c background [22]. However, it became apparent following further investigation that these immunized animals did not have antibodies capable of inducing ADRB against PyPEMS. ROS production by PMNs has been widely tested in the literature using such methodology [43, 51, 73–75], but these data highlight the importance of using native parasites in such an assay set-up. Previous work on ADCI activity suggests that it is not overly surprising that C-terminal MSP1 (MSP1_42_) immunization did not induce antiparasite cellular activation, given that investigation of antibody-dependent monocyte activation has identified other antigenic targets, such as glutamate-rich protein [76], serine repeat protein [77], MSP3 [78], and MSP1 block 2 [79]. As a result of the likely similarity in antibody action between ADCI and ADRB, the aforementioned targets may also represent those playing dominant roles in eliciting anti-Mz ADRB activity. The lack of detectable anti-PyPEMS ADRB activity post-PyMSP1_42_ vaccination was thus in agreement with the challenge data showing no bearing on an initial challenge in the knockout mice, and moreover, these data would suggest that the mechanism by which the PyMSP1_42_ vaccine protects is not FcR-mediated. Importantly, these data also suggested that anti-PyMSP1_19_ IgG responses may not be sufficient to induce ADRB activity, which would make sense, given the closeness of this moiety to the parasite surface and the fact the antigen may not be accessible to antibodies until the MSP1 molecule is processed during RBCs' invasion [80, 81]. However, it remained possible that throughout the course of a challenge infection, the animal could acquire de novo antiparasite antibodies capable of inducing ADRB, but this would be unlikely, given the short infection period investigated here with the highly lethal YM strain of P. yoelii. We thus switched to the use of a nonlethal model to assess postchallenge antibody responses.

In this case, following a nonlethal strain 17XNL primary infection, almost 50% of mice acquired anti-PyPEMS serum ADRB activity. This induction was irrespective of prior PyMSP1_42_ vaccination status and did not correlate with antibody titers against PyMSP1_19_ or PyMSP1_33_, again suggesting the activity to be independent of this antigen. However, it was then possible to reassess the importance of FcR and anti-PyPEMS ADRB activity in protection against secondary P. yoelii challenge. In this case, when mice were depleted of neutrophils, they were still able to control parasitemia, suggesting that these cells are not key effectors in controlling secondary infection in the P. yoelii model, despite measurable ADRB activity. Nevertheless, data from nonvaccinated γ^−/−^ mice in the same experiment alluded to a contribution of FcR-mediated signaling in controlling secondary exposure to P. yoelii malaria in mice and the possibility that this efficacy is mediated through a non-neutrophil cell group. There have been numerous reports of monocytes playing an important role in parasite killing through ADCI [9, 14, 16, 82, 83] or phagocytosis of infected RBCs. It would seem possible that in the absence of vaccine-induced responses against PyMSP1_42_, other antigen specificities acting against the Mz and/or infected RBC may play a more dominant role in effective immunity.

Overall, our data support the evidence that P. yoelii malaria infection is controlled largely by FcR-independent activity [18], especially given that both immunized and nonimmunized γ^−/−^ mice performed better than WT littermate controls in the challenge experiments. Interestingly, ADRB serum activity was again enhanced by secondary infection (with a likely increased contribution from anti-PyMSP1_33_ antibodies). Although no association was established with protection against P. yoelii, these data suggest that other antigen targets of anti-PEMS ADRB activity remain to be established. They also suggest that this activity increases with repeated malaria exposure, thus adding weight to the original report of association of this activity with clinical immunity against P. falciparum [24].

The development of new, functional antibody assays remains vital for preclinical malaria vaccine development. With relative ease, the principles of this assay could be transferred to assessment of ADRB activity using P. falciparum PEMS and human neutrophils, thus overcoming the limitations in extrapolating results from P. yoelii challenges in mice to humans and P. falciparum. Such research should provide important, new avenues for blood-stage malaria vaccines, complementing the development of GIA-based vaccines; however, further studies will be required to address whether this mechanism is directly contributing to immunity in humans, unlike in this mouse model.

## Supplementary Material

Supplemental Data
