# Supplementary material for: Assessment of antibody-dependent respiratory burst activity from mouse neutrophils on Plasmodium yoelii malaria challenge outcome
Source: J Leukoc Biol. 2014 Feb;95(2):369–82. doi: 10.1189/jlb.0513274 (PMC3896657; doi:10.1189/jlb.0513274)
Supplement: Supplemental Data [file supp_95_2_369__index.html]

Assessment of antibody-dependent respiratory burst activity from mouse neutrophils on Plasmodium yoelii malaria challenge outcome — Supplemental Data 

# Assessment of antibody-dependent respiratory burst activity from mouse neutrophils on Plasmodium yoelii malaria challenge outcome

## Supplemental Data

**Files in this Data Supplement:**

- Supplemental Data - (*jlb.0513274SuppData.doc; 326 KB*)
