# Supplementary material for: Assessment of antibody-dependent respiratory burst activity from mouse neutrophils on Plasmodium yoelii malaria challenge outcome
Source: J Leukoc Biol. 2014 Feb;95(2):369–82. doi: 10.1189/jlb.0513274 (PMC3896657; doi:10.1189/jlb.0513274)
Supplement: Supplemental Data [file supp_jlb.0513274_jlb.0513274SuppData.doc]

# Supplementary Material

### Supplementary Figure 1: Confirming PyMSP1 antibody depletion protocol

ADRB induction was assessed by BALB/c PMNs on either **(A)** PyMSP1_19_ or **(B)** PyMSP1_33_ coated plates in response to sera from BALB/c mice immunized with Ad-M PyMSP1_42_ which was pre-incubated with PBS (no depletion; black), or depleted of PyMSP1_19_ (grey) or PyMSP1_33_ (white) antibodies. Bars represent the mean of two replicates for each sample.

SSC-A

FSC-A

CD11b

Ly6C

A

B

### Supplementary Figure 2: Gating strategy

Bone marrow was extracted from the femurs and tibias of mice, and PMNs isolated on Percoll density gradients. Cells were surface stained with anti-CD11b, anti-Ly6C, and anti-CD8α. Events were acquired until 10,000 (in the case of blood samples) or 100,000 (spleen samples) CD8^+^ events had been measured. **(A)** Granulocytes, were gated by forward (FSC-A) and side scatter (SSC-A) profiles. **(B)** PMNs were then defined as the Ly6C^int^ CD11b^+^ population.

### Supplementary Figure 3: PyPEMS ADRB assay reproducibility

ADRB induction against PyPEMS was assessed using BALB/c PMNs in response to serum from a naïve BALB/c mouse, or one previously challenged with 10^6^ Py17XNL pRBCs. ADRB activity was assessed on four separate days using four different PMN donors to assess inter-assay variability. Bars represent the mean of two assay replicates for each sample.
